# Evidence Supports Tradition: The *in Vitro* Effects of Roman Chamomile on Smooth Muscles

**DOI:** 10.3389/fphar.2018.00323

**Published:** 2018-04-06

**Authors:** Zsolt Sándor, Javad Mottaghipisheh, Katalin Veres, Judit Hohmann, Tímea Bencsik, Attila Horváth, Dezső Kelemen, Róbert Papp, Loránd Barthó, Dezső Csupor

**Affiliations:** ^1^Department of Pharmacology and Pharmacotherapy, University of Pécs, Medical School, Pécs, Hungary; ^2^Department of Pharmacognosy, University of Szeged, Szeged, Hungary; ^3^Department of Pharmacognosy, University of Pécs, Pécs, Hungary; ^4^Department of Surgery, Clinical Center, University of Pécs, Medical School, Pécs, Hungary; ^5^Interdisciplinary Centre for Natural Products, University of Szeged, Szeged, Hungary

**Keywords:** Roman chamomile, *Chamaemelum nobile*, Asteraceae, organ bath experiment, gastrointestinal preparations, spasmolytic effect, contractile action

## Abstract

The dried flowers of *Chamaemelum nobile* (L.) All. have been used in traditional medicine for different conditions related to the spasm of the gastrointestinal system. However, there have been no experimental studies to support the smooth muscle relaxant effect of this plant. The aim of our research was to assess the effects of the hydroethanolic extract of Roman chamomile, its fractions, four of its flavonoids (apigenin, luteolin, hispidulin, and eupafolin), and its essential oil on smooth muscles. The phytochemical compositions of the extract and its fractions were characterized and quantified by HPLC-DAD, the essential oil was characterized by GC and GC-MS. Neuronally mediated and smooth muscle effects were tested in isolated organ bath experiments on guinea pig, rat, and human smooth muscle preparations. The crude herbal extract induced an immediate, moderate, and transient contraction of guinea pig ileum via the activation of cholinergic neurons of the gut wall. Purinoceptor and serotonin receptor antagonists did not influence this effect. The more sustained relaxant effect of the extract, measured after pre-contraction of the preparations, was remarkable and was not affected by an adrenergic beta receptor antagonist. The smooth muscle-relaxant activity was found to be associated with the flavonoid content of the fractions. The essential oil showed only the relaxant effect, but no contracting activity. The smooth muscle-relaxant effect was also detected on rat gastrointestinal tissues, as well as on strip preparations of human small intestine. These results suggest that Roman chamomile extract has a direct and prolonged smooth muscle-relaxant effect on guinea pig ileum which is related to its flavonoid content. In some preparations, a transient stimulation of enteric cholinergic motoneurons was also detected. The essential oil also had a remarkable smooth muscle relaxant effect in this setting. Similar relaxant effects were also detected on other visceral preparations, including human jejunum. This is the first report on the activity of Roman chamomile on smooth muscles that may reassure the rationale of the traditional use of this plant in spasmodic gastrointestinal disorders.

## Introduction

*Chamaemelum nobile* (L.) All. (Asteraceae), widely known as Roman chamomile, is a perennial herb native to South-Western Europe, but it is cultivated as a medicinal plant all over Europe and in Africa as well. Dried flowers of the cultivated, double-flowered variety of the species are official in the European Pharmacopoeia (European Pharmacopoeia, 2008). Incorporating the plant in traditional herbal medicinal products has been acknowledged by the European Medicines Agency. The comminuted herbal substance (as tea) and a liquid extract of the plant (extraction solvent: ethanol 70% *v/v*) may be used for the symptomatic treatment of mild, spasmodic gastrointestinal complaints including bloating and flatulence. The British Herbal Pharmacopoeia indicates its use as carminative, anti-emetic, antispasmodic, and sedative (British Herbal Pharmacopoeia, 1971).

Roman chamomile (RC) has been used as a medicinal plant from the middle ages. The cultivation of the plant started in England in the 16th century (Hiller and Melzig, 1999). The double variety of the flower, which now serves as the main commercial drug, has certainly been known since the 18th century (Evans, 1989). The plant gained the name “nobile” (Latin, noble) to illustrate its superior therapeutic efficacy over *Matricaria recutita* L. (German chamomile) (Hiller and Melzig, 1999). The plant was first listed in the Pharmacopoeia of Württemberg (1741) as a carminative, painkiller, diuretic, and digestive aid (Lukacs, 1990). In the folk medicine of different regions of Europe, RC has been used for numerous conditions, including dyspepsia, flatulence, nausea and vomiting, anorexia, vomiting of pregnancy, dysmenorrhoea, and specifically for gastrointestinal cramps and flatulent dyspepsia associated with mental stress (Augustin et al., 1948; Rápóti and Romváry, 1974; Melegari et al., 1988; Rossi et al., 1988; Bradley, 1992). In the Mediterranean region, RC tea is consumed to improve appetite and also after meal to prevent indigestion (Rivera and Obon, 1995; Menendez-Baceta et al., 2014; Alarcün et al., 2015). Traditional use of RC is largely related to its supposed smooth muscle-relaxant activity.

The majority of the secondary metabolites described from the plant belong to the aliphatic esters (essential oil) (Fauconnier et al., 1996), sesquiterpene lactones (Bisset, 1994) and flavonoids (Herisset et al., 1971, 1973; Abou-Zied and Rizk, 1973; Pietta et al., 1991). The polysaccharide content of the dried flower is noteworthy, 3.9% (Lukacs, 1990). The supposed smooth muscle-relaxant activity of the plant might be attributed to its flavonoid content. Apigenin and luteolin possess remarkable smooth muscle relaxant effects on guinea pig ileum (Lemmens-Gruber et al., 2006).

Although several studies on the bioactivities of RC are available, the majority of these studies were carried out using the essential oil, which is not used medicinally, or the observed activities are not related to the traditional use of the plant. Several studies demonstrate the antimicrobial effects of RC essential oil against different bacterial and fungal strains (Hänsel et al., 1993; Piccaglia et al., 1993; Chao et al., 2000; Bail et al., 2009), and antifungal activity was demonstrated also for the aqueous extracts of RC (Magro et al., 2006). The anti-inflammatory capacity and heat shock protein modulating effects of the flavonoids apigenin and quercetin, as well as the anti-inflammatory activities of α-bisabolol, guajazulene, and chamazulene have been reported in preclinical studies (Viola et al., 1995; Baghalian et al., 2008, 2011; Hernández-Ceruelos et al., 2010). The polysaccharides of RC exerted antiphlogistic effect *in vivo* (Lukacs, 1990).

Although the use of RC extract for gastrointestinal problems seems to be related to its presumptive smooth muscle-relaxant effect, interestingly no *in vitro* or *in vivo* studies have been carried out so far to assess this bioactivity. However, in an *in vitro* study an aqueous extract of *C. nobile* was demonstrated to induce a vasorelaxant effect through the NO-cGMP pathway or possibly through a combination of Ca^2+^ channel inhibition plus NO-modulating and phosphodiesterase inhibitory mechanisms. After the oral administration of RC aqueous extract, significant hypotensive effect was observed in an animal study on spontaneously hypertensive rats (Zeggwagh et al., 2009), which may be related to the flavonoid content of the plant (Jouad et al., 2001). The clinical efficacy of orally applied preparations has not been studied yet, only the effects of external application (Schrader et al., 1997) and aromatherapeutic use (Wilkinson et al., 1999) have been reported.

The aim of the current study was to assess the effects of a hydroethanolic RC extract, its fractions and essential oil on gastrointestinal and urogenital smooth muscles, including preparations of human jejunum, in order to clarify the rationale for the use of this plant for smooth muscle relaxation. Experiments were carried out with an extract conforming to the monograph of the European Medicines Agency (see in section “Preparation of Herbal Extracts and Isolation of Reference Standards”), and also with the fractions of this extract and with the essential oil of the plant.

## Materials and Methods

### Plant Material

Roman chamomile flowers were purchased from Pál Bobvos (Hungary). The identity of the plant material was confirmed according to the requirements of the European Pharmacopoeia. A voucher specimen is stored for verification purposes in the herbarium of the Department of Pharmacognosy, University of Szeged. RC essential oil was purchased from Aromax Ltd. (Hungary).

### Chemicals

For the pharmacological experiments the following drugs were used: atropine sulfate (Sigma), α,β-methylene ATP lithium salt (Tocris), capsaicin, histamine dihydrochloride, indo-methacin, isoprenaline hydrochloride, N^G^-nitro-L-arginine, papaverine hydrochloride, prostaglandin F_2α_ (Sigma), methysergide (Sandoz), (±)-propranolol hydrochloride (Sigma), pyridoxalphosphate-6-azophenyl-2′,4′-disulfonic acid tetrasodium salt (PPADS), tetrodotoxin (Tocris), 8-amino-7-chloro-2,3-dihydro-1,4-benzodioxan-5-carboxylic acid,1′-butyl-4′-piperidinylmethyl ester (SB204070), N-(1-azabicyclo [2.2.2]oct-3-yl)-6-chloro-4-methyl-3-oxo-3,4-dihydro-2H-1,4-benzoxazine-8-carboxamide hydrochloride (Y25130) (all from Tocris). For dissolving and diluting of these drugs the following solvents were used: 96% ethanol for capsaicin, indomethacin, and prostaglandin F_2α_, DMSO for SB204070 and isotonic NaCl solution for the rest of the drugs. Solutions in organic solvents were administered at a maximum of 1 μl/ml, aqueous solutions at 1 or 3 μl/ml bathing solution.

Millipore Direct-Q UV3 clarifier (Molsheim, France) was used to produce purified water for the HPLC measurements. Methanol and ethanol (LiChrosolv^®^ HPLC grade) was obtained from Merck (Darmstadt, Germany). For chromatographic separation, polyamide (ICN Polyamide for Column Chromatography), Sephadex LH-20 (25–100 μm, Pharmacia Fine Chemicals), SiO_2_ (Silica gel 60 G, 15 μm, Merck), and SiO_2_ plates (20 cm × 20 cm Silica gel 60 F_254_, Merck) were used.

### Preparation of Herbal Extracts and Isolation of Reference Standards

For the pharmacological experiments, RC crude extract was prepared according to the description of the European Medicines Agency monograph (EMA-HMPC, 2012). Ten grams of plant material was extracted with 70% EtOH (3 × 100 ml) via ultrasonic bath, evaporated in vacuum and lyophilized (yield, 3.169%). Part of the RC crude extract was fractionated to gain RC extract fractions with different compositions for further experiments. Vacuum liquid chromatography on polyamide with elution by MeOH-H_2_O (20:80, 40:60, 60:40, 80:20, 100:0) was used to gain fractions from the crude extract as follows: F20, F40, F60, F80, and F100.

From the methanolic extract of 200 g RC flowers, four marker compounds (used as reference standards in further experiments) were isolated using medium pressure liquid chromatography (SiO_2_ as stationary phase), gel chromatography (Sephadex LH-20), preparative HPLC (RP), and preparative TLC (SiO_2_). The purity and identity of these compounds were analyzed by NMR. NMR spectra were recorded in MeOD on a Bruker Avance DRX 500 spectrometer (Bruker, Fallanden, Switzerland) at 500 MHz (^1^H) or 125 MHz (^13^C).

### HPLC Experiments

HPLC experiments were carried out on a Shimadzu LC-20AD Liquid Chromatograph (SPD-M20A diode array detector, CBM-20A controller, SIL-20AC_HT_ autosampler, DGU-20A_5R_ degasser unit, CTO-20AC column oven) using a Kinetex 5 μm C-18 100A (150 mm × 4.6 mm) with a gradient of 0,01% trifluoroacetic acid in H_2_O (A) and acetonitrile (B) as follows: 0–5 min 25% B, 14 min 28% B, 15 min 70% B, 16 min 70% B, 16.5 min 25% B, and 20 min 25% B. The flow was 1.2 ml/min, column oven temperature was 55°C. Detection was carried out within the range of 190–800 nm. For quantification, chromatograms were integrated at 344 nm. The reference standards and the evaporated extracts were dissolved in MeOH, filtered through a PTFE syringe filter and injected in volumes of 5 or 10 μl. Calibration curves were established for all the four reference standards.

### GC and GC-MS Experiments

The GC analysis was carried out with an HP 5890 Series II gas chromatograph (FID), using a 30 m × 0.35 mm × 0.25 μm HP-5 fused silica capillary column. The temperature program ranged from 60°C to 210°C at 3°C min^-1^, and from 210°C to 250°C (2 min hold) at 5°C min^-1^. The detector and injector temperature was set to 250°C and the carrier gas was N_2_, with split sample introduction. Quantities of the individual components of the essential oil were expressed as the percent of the peak area relative to the total peak area from the GC/FID analysis.

The GC-MS analysis was performed with a Finninan GCQ ion trap bench-top mass spectrometer. All conditions were as above except that the carrier gas was He at a linear velocity of 31.9 cm s^-1^ and the capillary column was DB-5MS (30 m × 0.25 mm × 0.25 μm). The positive ion electron ionization mode was used, with ionization energy of 70 eV, and the mass range of 40–400 amu.

Identification of the compounds was based on comparisons with published MS data (Adams, 2007) and with a computer library search (the database was delivered together with the instrument) and also by comparing their retention indices with literature values (Adams, 2007). Retention indices were calculated against C8–C32 *n*-alkanes on a CB-5 MS column (Kovats, 1965). A mixture of aliphatic hydrocarbons was injected in hexane (Sigma-Aldrich, St. Louis, MO, United States) by using the same temperature program that was used for analyzing the essential oil.

### Guinea Pig and Rat Preparations

Guinea pigs (short-haired, colored, 350–450 g) or Wistar rats (220–300 g) of either sex were killed by a blow to the head and exsanguination. Three centimeter segments of the ileum or distal colon were placed into the organ bath in a vertical position, under a constant tension of 7 mN. Longitudinal strip preparations (approximately 2–3 cm in length) of guinea pig urinary bladder or rat gastric fundus were fixed under a tension of 5 mN.

This study was carried out in accordance with University guidelines. The protocol was approved by the Animal Welfare Committee, University of Pécs (registration number, BA02/2000-1/2012) with the understanding that isolated organ studies after stunning the animal cannot be regarded as animal experiments.

### Organ Bath Experiments

Smooth muscle preparations were used in a traditional organ bath arrangement. The preparations were suspended in Krebs–Henseleit solution of 5 or 7 ml, kept at 37°C, and aerated with a mixture of 95% O_2_ and 5% CO_2_. The solution contained 119 mM NaCl, 25 mM NaHCO_3,_ 2.5 mM KCl, 1.5 mM MgSO_4_, 2.5 mM CaCl_2_, 1.2 mM KH_2_PO_4_, and 11 mM glucose (pH = 7.4). Movements of the preparations were recorded with isotonic transducers (Hugo Sachs Elektronik-Harvard Apparatus, March, Germany) on ink writers or stored online on a personal computer.

The guinea pig ileum is a preparation of a low spontaneous tone. In part of the experiments the effects of RC extract, its fractions, essential oil, or flavonoids were studied at basal tone. All experiments commenced with determining the maximal longitudinal spasm by adding histamine (10 μM) to the organ bath for 2 min. This response served as reference for determining the relative amplitude of any contractile effect that the samples to be tested might have had.

In another set of experiments the possible relaxant effect of the materials was tested. For this reason, a tonic, approximately half-maximal contraction of the ileum was provoked by a moderate concentration of histamine (0.5 μM, added for 15 min). In preliminary experiments, no pretreatment was used. In the bulk of the experiments, however, the muscarinic receptor antagonist atropine, as well as tetrodotoxin, an inhibitor of nerve axonal conduction (blocker of voltage-sensitive Na^+^ channels) was added prior to the histamine administration. This was done to avoid the interference with a possible contractile effect of the material to be examined, i.e., to create a methodologically clear situation.

All experiments commenced after an equilibration period of 45 min. Relaxant effects were studied on pre-contracted preparations (see below). Maximal spasm of the tissue was evoked to serve as a basis for the comparisons to evaluate the responses obtained by our test materials. The materials examined were administered in a single concentration (i.e., in a non-cumulative manner).

### Human Jejunal Preparations

Macroscopically intact segments of human jejunum, removed during the surgical treatment of pancreatic cancer were used. Strips of either longitudinal or circular orientation were prepared (the mucosa and submucosa were removed) and fixed as described above, under a tension of 10 mN. Freeze-dried extracts of RC, as well as the essential oil of the plant were mixed in and further diluted with DMSO. The amounts of DMSO administered to the jejunum preparations are indicated in the Section “Results”.

This study was carried out in accordance with the Declaration of Helsinki and the guidelines set by the Research Ethics Committee, Scientific Council of the Ministry of Health, Hungary. This Committee agreed to the use of discarded human tissue for the experimental work. The protocol was approved by the ETT-TUKEB (Scientific Council of the Ministry of Health, Research Ethical Committee) [No. 17861-0/2010-1018EKU (749/PI/10)]. All subjects gave written informed consent in accordance with the Declaration of Helsinki.

### Statistical Analysis

Results of the pharmacological experiments are given as mean ± SEM, with *N* denoting the number of preparations used for testing efficacy. At most 2 preparations from the same animal were used for each type of experiment. Results are provided in relative values, where 100% contraction means maximal spasm of the preparations from the basal tone and, in pre-contracted preparations, 100% relaxation means relaxation of the preparation to the level before adding the precontracting agent. The Mann–Whitney *U* test was used for the comparisons of two independent groups, more than two independent samples were compared by the Kruskal–Wallis test, and two dependent samples were compared by the Wilcoxon test. The statistical program GraphPad Prism 5 was used throughout. A probability of *P* < 0.05 or less indicated statistical significance.

## Results

### Chemical Characterization of RC Extracts

**FIGURE 1:**
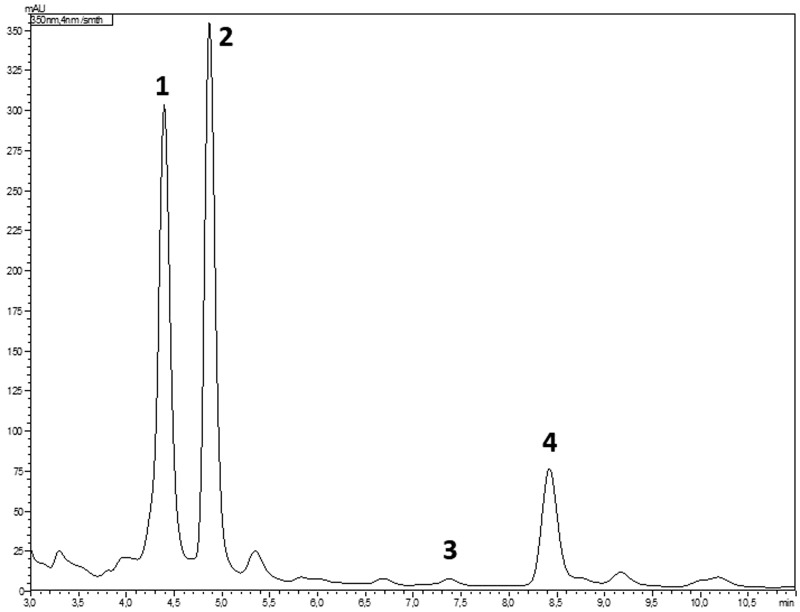
HPLC chromatogram of the crude RC extract with the peaks of luteolin (1), eupafolin (2), apigenin (3), and hispidulin (4) (344 nm).

In the HPLC retention time range of 4–9 min, four characteristic peaks were detected in the crude RC extract and its fractions F40–F100. The corresponding components were isolated from the plant material and were identified as the flavonoids apigenin, eupafolin, hispidulin, and luteolin by ^1^H and ^13^C NMR experiments (see Supplementary Figures S1–S6). These compounds were further used as reference standards to characterize the RC extracts. The identification of reference compounds in different extracts was based on matching the retention times and UV spectra. Retention times of luteolin, eupafolin, apigenin, and hispidulin were 4.5, 5.0, 7.5, and 8.5 min, respectively (**Figure 1**). The baseline separation of these compounds allowed their reliable quantification in different extracts.

The crude RC extract contained eupafolin as the main flavonoid, followed by luteolin, hispidulin, and apigenin (**Table 1**). The fractionation on polyamide resulted in subfractions F20–F100 with different compositions, as demonstrated by the differences in their flavonoid content. In F20, the quantities of flavonoids were below the level of quantification. The flavonoid content of the fractions increased with increasing MeOH content of the eluting solvent. The highest flavonoid levels were measured in F80, except for luteolin and apigenin, which were mainly concentrated in F100.

**Table 1:** Flavonoid content of RC crude extract and its fractions as determined by HPLC.

Sample	Luteolin (mg/g extract)	Eupafolin (mg/g extract)	Apigenin (mg/g extract)	Hispidulin (mg/g extract)
Crude extract	4.617 ± 0.616	18.756 ± 2.121	0.298 ± 0.027	1.584 ± 0.181
F20	Not detected	Not detected	Not detected	Not detected
F40	0.578 ± 0.001	1.800 ± 0.001	0.179 ± 0.001	0.231 ±< 0.001
F60	1.904 ± 0.001	62.591 ± 0.025	0.151 ±< 0.001	5.951 ± 0.004
F80	22.605 ± 0.001	223.488 ± 0.036	0.859 ±< 0.001	17.060 ± 0.006
F100	55.305 ± 0.002	150.206 ± 0.005	2.055 ±< 0.001	4.983 ±< 0.001

### Chemical Characterization of RC Essential Oil

Based on their retention times and mass spectrometric data, methallyl angelate, 3-methyl pentyl angelate, and 3-methylamyl isobutyrate were identified as the major constituents of RC essential oil (19.0, 18.2, and 10.4%, respectively) (**Table 2**). The identified components comprised 97% of the essential oil. Previous articles reported isobutyl angelate as the major constituent of RC essential oil (21.6–38.5%), followed by 2-methylbutyl angelate (11.6–20.3%) (Antonelli and Fabbri, 1998; Farkas et al., 2003; Omidbaigi et al., 2003, 2004; Bail et al., 2009), and propyl tiglate (10.8–13.1%) (Omidbaigi et al., 2003, 2004) or isobutyl isobutyrate (3.3%) (Bail et al., 2009) or 2-butenyl angelate (7.9–8.4%) (Antonelli and Fabbri, 1998) or 2-methyl-2-propenyl angelate (9.1%) (Farkas et al., 2003). However, several commercial samples have similar compositions to the sample analyzed by us, with methallyl angelate and 3-methyl pentyl angelate as the major components.

**Table 2:** Chemical composition of RC essential oil.

Compounds^a^	RI^b^	% in samples
Methyl tiglate	867	tr
*n*-Hexanol	875	1.1
2-Methylbutyl acetate	897	0.4
Isobutyl isobutyrate	925	0.8
Acetonyl acetone	932	0.7
α-Pinene	935	2.4
Camphene + allyl methacrylate	958	0.6
Thuja-2,4(10)-diene	960	1.1
Isoamyl propionate	966	tr
β-Pinene	969	0.3
Myrcene	973	0.6
Propyl angelate	993	1.1
Isobutyl 2-methylbutyrate	998	tr
Isoamyl isobutyrate	1,004	1.5
2-Methylbutyl isobutyrate	1,006	2.7
1,8-Cineol	1,035	tr
Isoamyl methacrylate	1,037	1.1
Isobutyl angelate	1,058	4.9
Methallyl angelate	1,068	19.0
2-Butenyl angelate	1,119	tr
3-Methylamyl isobutyrate	1,122	10.4
3-Methylamyl methacrylate	1,150	6.6
*trans*-Pinocarveol + isoamyl angelate	1,153	8.6
2-Methylbutyl angelate	1,168	8.3
Pinocarvone	1,177	3.9
Prenyl angelate	1,213	1.5
Myrtenal	1,217	1.2
3-Methyl pentyl angelate	1,264	18.2
**Identified components**		**97.0**

^a^*Compounds listed in sequence of elution from a DB-5 MS column. ^*b*^Retention indices calculated against C8 to C32 n-alkanes on a DB-5MS column. tr, in traces.*

### Effects on Guinea Pig Ileum

**FIGURE 2:**
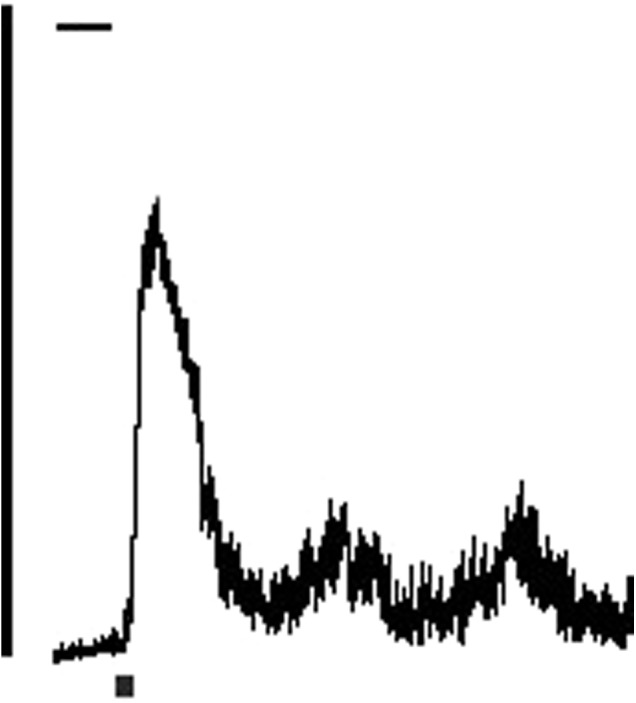
Contractile effect of RC extract (60 μg/ml, added at the square symbol) on the guinea pig ileum. Calibrations, vertical: 50% of the maximal longitudinal spasm evoked by histamine (10 μM), horizontal: 1 min.

The crude RC extract induced a transient longitudinal contraction on guinea pig ileum preparations (**Figure 2**). The amplitudes of the contractions were related to the maximal longitudinal spasm evoked with 10 μM of histamine at the beginning of the experiments. The threshold concentration for this effect was equal to or below 20 μg/ml of the extract (which was the lowest concentration tested) and reached a plateau with 60 and 200 μg/ml. Quantitative results were as follows (expressed as % of the maximal spasm): 18.8 ± 3.1% at 20 μg/ml (*N* = 6), 40.1 ± 3.3% at 60 μg/ml (*N* = 11), and 36.3 ± 4.9% at 200 μg/ml (*N* = 7). A second administration of the same concentration after a 40-min washout period usually had a qualitatively similar effect. Yet, because of variable reproducibility, we examined the effects on separate preparations with only one administration of the extract. The solvent of the extract (DMSO; 0.3 or 1 μl/ml) caused no or minimal contraction (on average, 0 and 2%, respectively; *N* = 12).

The 60 μg/ml concentration of the extract was used for pharmacological analysis. Both atropine (0.5 μM), an antagonist of acetylcholine at the muscarinic receptors and tetrodotoxin (0.5 μM), an inhibitor of voltage-sensitive Na^+^ channels (hence, of neuronal axonal conduction) inhibited the contractile effect of RC crude extract. In contrast, the purinoceptor antagonist PPADS (50 μM) or a combination of serotonin (5-HT) receptor antagonists methysergide (0.3 μM), SB204070 (1 μM) and Y25130 (1 μM) failed to influence the contractile effect of the extract (**Table 3**). This combination of 5-HT antagonists is suitable for blocking the contractile effect of 5-HT (Sandor et al., 2016).

**Table 3:** Effects of drugs on the contractile response to RC crude extract (60 μg/ml) on guinea pig small intestine (mean ± SEM).

Pretreatment	Contraction (% of maximal spasm)	*N*
No pretreatment (control)	40.1 ± 3.3	11
Tetrodotoxin (0.5 μM)	15.0 ± 1.8^∗^	6
Atropine (0.5 μM)	8.2 ± 2.0^∗^	6
PPADS (50 μM)	49.7 ± 2.7	9
5-HT receptor antagonists^#^	52.1 ± 3.0	11
Solvent for capsaicin	51.1 ± 5.2	7
Capsaicin^&^	46.9 ± 6.0	9
Solvent for indomethacin	46.4 ± 4.0	6
Indomethacin (3 μM)	31.2 ± 5.6^∗^	10

*Values significantly different from the respective control group are indicated by asterisks (^∗^). ^#^methysergide (0.3 μM), SB204070 (1 μM) and Y25130 (1 μM). ^&^10 μM of capsaicin for 10 min, followed by a 60-min washout period.*

The functional blockade of capsaicin-sensitive neurons did not inhibit the contractile effect of the RC extract (Barthó et al., 2004) as compared to time-matched, solvent-treated controls (**Table 3**). The cyclooxygenase inhibitor indomethacin (3 μM) moderately but significantly inhibited the contraction in response to the RC extract.

Fractions of the RC extract were also tested for contracting activity. Fraction F20 induced a moderate contraction (approximately 20% of the maximum) (**Table 4**). Similar results were obtained with F40, F60, F80, and F100, although the extent of contraction tended to decline with F60, F80, and F100.

**Table 4:** Contractile effects of RC extract fractions on guinea pig ileum (longitudinally oriented preparations, mean ± SEM).

Bath concentration	Contraction (% of the maximal spasm)	*N*
**F20**		
2 μg/ml	5.2 ± 2.4%	5
20 μg/ml	18.2 ± 6.1%	5
60 μg/ml	18.5 ± 4.4%	6
200 μg/ml	22.2 ± 3.1%^∗^	5
**F40**		
2 μg/ml	2.5 ± 1.2%	5
20 μg/ml	18.2 ± 5.9%	5
60 μg/ml	15.5 ± 3.3%	5
200 μg/ml	22.8 ± 8.4%^∗^	5
**F60**		
2 μg/ml	8.4 ± 1.3%	6
20 μg/ml	21.8 ± 6.4%^∗^	5
60 μg/ml	20.9 ± 8.2%^∗^	5
200 μg/ml	4.0 ± 2.7%	6
**F80**		
2 μg/ml	0.4 ± 0.4%	5
20 μg/ml	13.0 ± 3.2%	5
60 μg/ml	12.0 ± 4.0%	5
200 μg/ml	2.5 ± 1.5%	5
**F100**		
20 μg/ml	12.4 ± 2.1%	6
60 μg/ml	26.4 ± 5.8%^∗^	5
200 μg/ml	15.1 ± 2.7%	6

*Asterisks denote values significantly different from a pooled group of solvent effects (Kruskal–Wallis test for several unrelated samples).*

**FIGURE 3:**
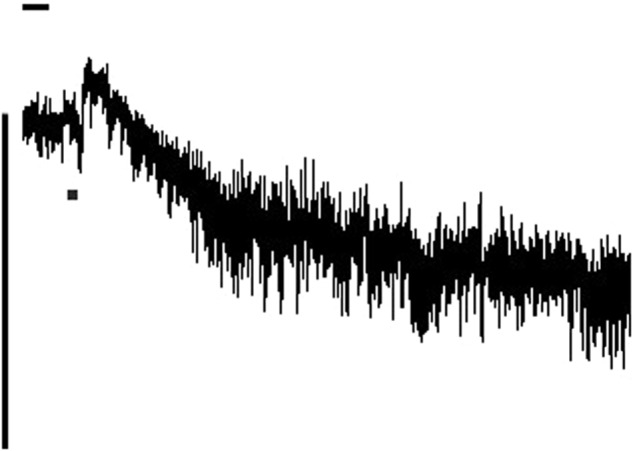
Long-lasting relaxant effect of RC extract (60 μg/ml, added at the square symbol) on the pre-contracted guinea pig ileum, in the presence of tetrodotoxin and atropine (0.5 μM each). Calibrations, vertical: 100% relaxation from the pre-contracted baseline, horizontal: 1 min.

Our experiments revealed the smooth muscle relaxing activity of RC extract, fractions and essential oil in this experimental setting. On histamine-precontracted preparations (treated with 0.5 μM histamine for 15 min, in the presence of atropine and tetrodotoxin, both 0.5 μM), concentration-dependent relaxation was observed in response to treatment with RC crude extract (60–200 μg/ml) (**Figure 3** and **Table 5**). The highest concentration tested induced full relaxation. The relaxation detected with the 20 μg/ml test sample did not exceed the changes evoked by the solvent itself (1 μl/ml DMSO; see below). In several cases the relaxation was preceded by a little contraction. The relaxation induced by the 60 μg/ml extract was not significantly altered by the adrenergic β-receptor antagonist propranolol (1 μM; 51.3 ± 7.8% relaxation, *N* = 6) or by the NO synthase inhibitor N^G^-nitro-L-arginine (100 μM; 47.4 ± 7.4% relaxation, *N* = 10) (both compared with the group indicated in **Table 5**). The solvent DMSO (0.3 or 1 μl/ml) had a slight relaxant effect in this experimental arrangement (histamine-precontracted ileum pretreated with atropine and tetrodotoxin); it amounted to 3.0 ± 1.6% at 0.3 μl/ml and 7.9 ± 1.4% at 1 μl/ml; *N* = 11 and 17, respectively.

**Table 5:** Relaxing effect of RC crude extract and essential oil on the pre-contracted ileum.

Bath concentration of RC crude extract	Relaxation (% of the maximum)	*N*
20 μg/ml	18 ± 5.2%	6
60 μg/ml	76.2 ± 8.5%^∗^	9
200 μg/ml	100%^∗^	5

**Bath concentration of RC essential oil**	**Relaxation %**	***N***

0.1 μg/ml	12.8 ± 3.5%	6
1 μg/ml	30.8 ± 5.9%^∗^	9
10 μg/ml	69.7 ± 5.6%^∗^	6

*Atropine and tetrodotoxin (0.5 μM each) were present in the medium in order to inhibit the contractile effect of the RC extract. Tonic submaximal contraction was evoked with histamine (0.5 μM for 15 min). Values are given in %, where 100% means full relaxation reaching the pre-histamine values (mean ± SEM). Asterisks denote values significantly different from a pooled group of solvent effects (0.3 or 1 μl/ml of DMSO, see text) (Kruskal–Wallis test for several unrelated samples).*

On histamine-precontracted, atropine- and tetrodotoxin-treated ileum, different fractions of RC extract showed distinct relaxant effects (**Table 6**). F20 and F40 (60 or 200 μg/ml) practically produced no relaxation (*N* = 5 for both concentrations). This observation refers to the potential role of flavonoids in the relaxant effect, and experiments with four flavonoids isolated from the plant material reassured this hypothesis. All the flavonoids exerted a dual effect on the guinea pig ileum, i.e., a short-lived contraction at basal tone and a long-lasting relaxation on the histamine-pre-contracted, atropine- and tetrodotoxin-pretreated preparations (**Table 7**). At a concentration of 1 μM, the flavonoids did not exert contractile activities, however, at 10 μM, a slight effect (34.6 ± 7.4% for hispidulin, 32.0 ± 3.1% for luteolin, 27.1 ± 4.2% for eupafolin, and 20.5 ± 5.1% for apigenin, *N* = 5 each) was observed. The relaxant activities at 2 μM ranged between 18.2 ± 5.4% and 24.2 ± 3.7%, whereas at 20 μM between 64.5 ± 4.1% and 81.9 ± 5.3%.

**Table 6:** Relaxant effects of RC extract fractions on pre-contracted guinea pig ileum (mean ± SEM).

Bath concentration	Relaxation %	*N*
**F60**		
20 μg/ml	18.7 ± 5.7%	5
60 μg/ml	96.0 ± 3.0%^∗^	6
200 μg/ml	93.5 ± 4.9%^∗^	6
**F80**		
20 μg/ml	47.2 ± 7.7%	5
60 μg/ml	93.0 ± 5.9%^∗^	6
200 μg/ml	100%^∗^	4
**F100**		
6 μg/ml	12.5%	4
20 μg/ml	61 ± 13.7%	5
60 μg/ml	69.4 ± 7.5%^∗^	6
200 μg/ml	100%^∗^	4

*100% relaxation denotes that tone returns to baseline. Asterisks denote values significantly different from a pooled group of solvent effects (0.3 or 1 μl/ml DMSO) (Kruskal–Wallis test for several unrelated samples).*

**Table 7:** Relaxant effects of flavonoids on pre-contracted guinea pig ileum (mean ± SEM).

Bath concentration	Relaxation %	*N*
**Hispidulin**		
2 μM	19.4 ± 3.5%	5
20 μM	64.5 ± 4.1%	6
**Luteolin**		
2 μM	19.6 ± 1.6%	5
20 μM	80.0 ±-4.5%	6
**Eupafolin**		
2 μM	18.2 ± 5.4%	5
20 μM	68.7 ± 6.8%	6
**Apigenin**		
2 μM	24.2 ± 3.7%	5
20 μM	81.9 ± 5.3%	6

*100% relaxation denotes that tone returns to baseline. The solvent contained a maximum of 10% DMSO and had no relaxant effect in the volumes used.*

The essential oil of RC (0.1, 1, 10, or 30 μg/ml) showed no contractile effect on the ileum (*N* = 6–8). RC oil (1 or 10 μg/ml) induced considerable relaxation on histamine-precontracted, atropine- and tetrodotoxin-pretreated preparations (**Table 5**). Similar results were obtained on preparations without atropine and tetrodotoxin pretreatment (*N* = 6–8, data not shown).

Papaverine was used as positive control. As with other experiments for studying relaxation, the drug was administered to histamine-precontracted, atropine- and tetrodotoxin-treated ileum preparations, in a non-cumulative manner. The relaxant responses obtained were as follows, 0.3 μM: 19.7 ± 4.7% (*N* = 6); 1 μM: 29.1 ± 3.5% (*N* = 6); 3 μM: 30.1 ± 4.8% (*N* = 7); 10 μM: 92.9 ± 4.2% (*N* = 6), where, as also elsewhere in this paper, relaxation to the pre-histamine baseline was taken as 100%.

### Effects on Guinea Pig Urinary Bladder

**FIGURE 4:**
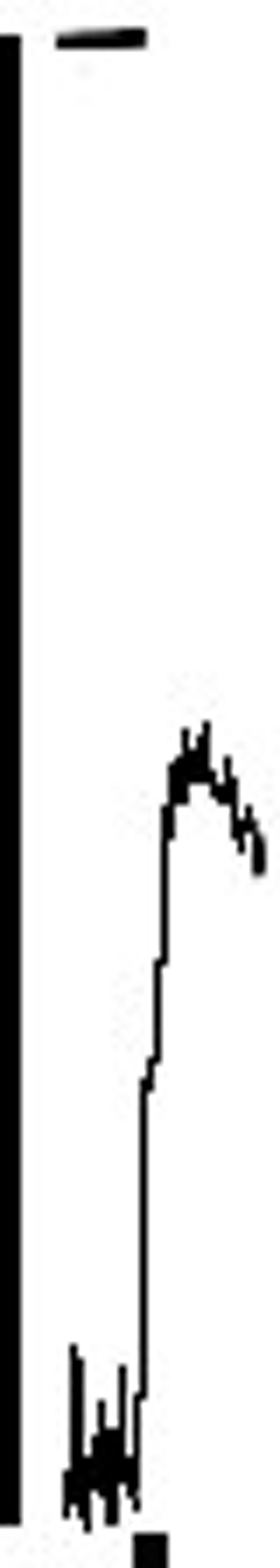
Contraction, in response to 200 μg/ml RC extract (added at the square symbol), on the guinea pig urinary bladder detrusor strip. Calibrations, vertical: 50% of the maximal spasm in response to 100 mM KCl, horizontal: 1 min.

The effects of the RC extract and the essential oil were tested on guinea pig urinary bladder strips as well. RC crude extract evoked concentration-dependent contraction at 20 and 200 μg/ml (**Table 8** and **Figure 4** for 200 μg/ml). The solvent of the extract had no contractile effect on the bladder (*N* = 4). The effect of the 200 μg/ml extract was diminished (approximately halved) by *in vitro* capsaicin pretreatment (**Table 8**). Contractile responses were compared to the maximal spasm evoked by 100 mM of KCl at the end of the experiment.

**Table 8:** Contractile effect of RC extract on guinea pig urinary bladder strip, without pretreatment and following treatment with capsaicin or its solvent (mean ± SEM).

Bath concentration of RC crude extract	Contraction (% of the maximal spasm)	*N*
20 μg/ml No pretreatment	8.0 ± 4.2%	7
200 μg/ml No pretreatment	20.0 ± 5.1%	7
200 μg/ml Ethanol pretreatment^#^	21.6 ± 4.9%	8
200 μg/ml Capsaicin (10 μM) pretreatment^&^	9.1 ± 1.3%^∗^	8

*^#^Solvent and time control for capsaicin. ^&^Capsaicin was added and incubated for 10 min. This was followed by a 90-min washout period. ^∗^Significantly different from the ethanol control.*

To study the relaxant effect of the crude extract, the bladder strips were sub-maximally pre-contracted with histamine (1–3 μM). Atropine and tetrodotoxin were present in the bathing fluid. Since excitatory purinergic mechanisms are present in the bladder, α,β-methylene ATP desensitization (10 + 10 μM for 10 min each) was also performed. RC crude extract had no effect at a concentration of 20 μg/ml, whereas a concentration of 200 μg/ml induced 58.3 ± 4.6% relaxation (*N* = 7 and 8, respectively). The solvent itself (1 μl/ml DMSO) exerted no effect.

Roman chamomile essential oil caused no contraction in a concentration of 10 μg/ml. Similarly to the extract, RC oil exerted a moderate relaxing effect on the pre-contracted bladder: 17.9 ± 6.1% and 34.1 ± 5.8% relaxation was evoked with 1 and 10 μg/ml, *N* = 7 and 6, respectively.

### Effects on Rat Gastrointestinal Preparations

The relaxant effect of RC essential oil was confirmed on longitudinally oriented preparations of the rat gastrointestinal tract. Rat whole ileum and distal colon preparations are characterized by high intrinsic tone, therefore no pre-contraction was needed. RC oil (10 μg/ml) induced relaxation (41.4 ± 7.4% and 21.8 ± 3.7%, *N* = 7 and 10, respectively, on these preparations, where 100% means the relaxant response to 3 μM isoprenaline, given at the end of the experiment). A concentration of 1 μg/ml was barely effective. No contractile effect was observed. Rat stomach fundus strips were pre-contracted with 0.1–0.3 μM acetylcholine. RC oil produced relaxation (on average 44 and 70.3% relaxation in response to concentrations of 1 and 10 μl/ml, *N* = 4 each, where 100% means relaxation to the pre-acetylcholine level).

### Effects on Human Jejunal Preparations

In untreated longitudinally oriented human jejunal preparations RC crude extract induced transient contraction amounting to 12.4 ± 3.5% and 30.5 ± 0.6% at 20 and 200 μg/ml concentrations, respectively (*N* = 8). Atropine- and tetrodotoxin-pretreated preparations were pre-contracted with PGF2α (1 or 2 μM). RC extract showed a concentration-dependent relaxant effect (25.8 ± 4.3% at 20 μg/ml, *N* = 8; 68.5 ± 8.5% at 60 μg/ml, *N* = 7; 50.8 ± 3.7% at 200 μg/ml, *N* = 9). RC essential oil produced no contraction. A lasting relaxant effect was detected on pre-contracted preparations (in the presence of atropine and tetrodotoxin). The relaxant effect reached 26.9 ± 5.2% at 1 μg/ml and 81.4 ± 8.2% at 10 μg/ml concentrations (*N* = 8 and 7, respectively).

In untreated circularly oriented human jejunal preparations the RC crude extract evoked transient contraction (43.4 ± 10% at 200 μg/ml, *N* = 5), while the 20 μg/ml concentration sample had a negligible effect (2.8% contraction on 5 preparations). RC extract induced relaxation in PGF_2α_-precontracted preparations (in the presence of atropine and tetrodotoxin). This effect reached 45% with 60 μg/ml concentration (*N* = 4), while a concentration of 200 μg/ml produced full relaxation (*N* = 6), where a relaxation to the pre-prostaglandin level was taken as 100%.

The solvent itself (1 μl/ml DMSO) did not evoke either contraction or relaxation on this preparation (*N* = 4–6).

## Discussion

The experiments presented here have been carried out with RC, which has been used traditionally for the symptomatic treatment of mild, spasmodic and other gastrointestinal complaints, however, the presumed smooth muscle-relaxant effect has not been confirmed experimentally. The aim of our study was to investigate the effect of a traditionally used extract of the plant, its fractions and the essential oil of the plant, as well as of four of its flavonoid components.

The phytochemical analysis of the extract revealed the presence of flavonoids in the plant, four of which (apigenin, luteolin, eupafolin, and hispidulin) were isolated and identified. The hydroethanolic extract was fractionated on polyamide to gain fractions with different flavonoid content in order to examine the role of these compounds in the effect on smooth muscles. The flavonoid content of the extract and fractions was analyzed by HPLC. The flavonoid content of the extract was fractionated successfully, fractions F80, F100 (and to some extent F60) containing higher amounts of flavonoids than the crude extracts. The flavonoid pattern of fractions also differed. The essential oil analyzed by us belongs to the chemotype characterized by the predominance of methallyl angelate.

Pharmacological experiments were carried out with the hydroethanolic extract, its fractions (F20, F40, F60, F80, and F100), four flavonoids (apigenin, eupafolin, hispidulin, and luteolin) and the essential oil of the plant on different smooth muscles *in vitro*. The hydroethanolic extract of RC has both stimulatory and relaxant effects on guinea pig ileum. The moderate, transient stimulatory activity results from the activation of cholinergic neurons, as confirmed by its inhibition by tetrodotoxin (an inhibitor of neuronal voltage-sensitive Na^+^ channels) and atropine (antagonist on muscarinic receptors for acetylcholine). Although capsaicin, a stimulant of a certain class of sensory receptors (through which it evokes a “local efferent” response) shows a similar cholinergic, neurogenic effect in guinea pig small intestine preparations (see Barthó et al., 2004 for review), a functional blockade of capsaicin-sensitive nerve endings by capsaicin pretreatment failed to inhibit the contractile action of RC extract. The contractile action of RC extract was moderately, yet significantly reduced by the cyclooxygenase inhibitor indomethacin, which may indicate a modulatory role of endogenous prostanoids. Other pharmacological inhibitors tested had no contraction-reducing effect, therefore it is proposed that neither endogenous serotonin, nor PPADS-sensitive purinergic mechanisms play a role in the excitatory action of RC extract. It should be noted that both serotonin and ATP or the P_2X_ receptor agonist α,β-methylene ATP are able to evoke cholinergic contractions in guinea pig ileum (Benko et al., 2005; Barthó et al., 2006; Sandor et al., 2016 for recent data).

Following the transient and mild-to-moderate stimulant effect, the RC crude extract exhibited a sustained relaxant effect on the guinea pig ileum in the presence of tetrodotoxin and atropine, and preliminary experiments indicated a similar relaxant effect also in the absence of atropine and tetrodotoxin. Yet, atropine and tetrodotoxin were included into these experiments for creating a methodologically clear situation, where an initial, neuronally mediated excitatory action would hardly interfere with the relaxant one. The concentrations of the extract causing relaxation were roughly the same as those causing contraction. Based on these data we propose that the site of action for the relaxant effect of RC is on the smooth muscle itself. Neither the adrenergic β-receptor antagonist propranolol nor the NO synthase inhibitor N^G^-nitro-L-arginine significantly reduced the relaxant effect of the RC extract, hence, no evidence was found for these mechanisms to be involved in the relaxant response. In fact, a direct relaxant effect of the extract was demonstrated on all gastrointestinal preparations tested, including human and rat gastrointestinal preparations, as well as in guinea pig bladder. In preparations with high intrinsic tone (rat ileum and rat colon), relaxant activity was practically the only response to be seen.

Different RC extract fractions evoked both contraction and relaxation on guinea pig ileum. While there was a clear-cut tendency correlation between the flavonoid content and the relaxant effect, in case of contractile action such correlation was not observed (on the contrary, fractions with lower flavonoid content exerted slightly higher contractile activities). Fractions with high flavonoid content (F60, F80, and F100) had remarkable relaxant effects, whereas fractions with no (F20) or low (F40) flavonoid content exerted no such activity. The pure flavonoids of the extract showed dual effects in the guinea pig ileum, much similarly to the crude extract. Moreover, there was no substantial difference between the potencies of the flavonoids. This means that any of these flavonoids may contribute to the sustained relaxant and the transient contractile effect of the extract. The essential oil of RC caused no contraction on the test preparations; nevertheless, a consistent relaxant effect was detected. This seems to indicate that (i) chemical components responsible for the stimulant effect may be absent in the essential oil; (ii) it may be that several types of compounds present in the extract are responsible for the smooth muscle-relaxant action. This point needs to be clarified in subsequent experiments.

The contraction of guinea pig bladder in response to the RC extract proved to be special, in that it was reduced by half following *in vitro* capsaicin pretreatment. This indicates that capsaicin-sensitive sensory nerves of the bladder wall are in some way involved in the excitatory effect of the RC extract (see Barthó et al., 2004). Nevertheless, the RC extract and oil also induced bladder relaxation in pre-contracted preparations.

Roman chamomile extract was effective in human jejunal preparations as well. Both an excitatory and a tetrodotoxin- and atropine-resistant relaxing effect were demonstrated, again, the smooth muscle relaxant one being more sustained than the excitatory one. Preliminary experiments have shown that this early contraction is reduced by atropine. Due to limited access to human tissue, no further analysis of these responses could be performed.

In terms of possible medical uses it may be noted that a combination of nerve-mediated, mild-to moderate excitatory effect and a smooth muscle-relaxant action may even be advantageous for some gastrointestinal problems, e.g., diminished peristaltic activity, while for spasms in the stomach or large intestine it is obviously the smooth muscle relaxing activity that offers benefits.

## Conclusion

Our results support the overall smooth muscle relaxant effect of a hydroethanolic RC extract and of the essential oil of RC. The predominant effect of the extract, its fractions and flavonoids is relaxation, being more sustained than the transient contraction observed in some cases. This activity is in correlation with the flavonoid content of the extract. Since the components of the essential oil are partly extracted with alcohols, the constituents of the oil also contribute to the overall effect of the hydroethanolic RC extract. The extract used in our experiments was prepared in accordance with the European Medicines Agency monograph for this plant, therefore our study contributes to the body of evidence relating the traditional use of this plant.

## Author Contributions

ZS, DK, TB, and RP carried out the organ bath experiments. JM, KV, and AH performed the phytochemical studies. JH, LB, and DC designed and co-ordinated the experiments and wrote the manuscript.

## Conflict of Interest Statement

The authors declare that the research was conducted in the absence of any commercial or financial relationships that could be construed as a potential conflict of interest.
