# Supplementary material for: Evidence Supports Tradition: The in Vitro Effects of Roman Chamomile on Smooth Muscles
Source: Front Pharmacol. 2018 Apr 6;9:323. doi: 10.3389/fphar.2018.00323 (PMC5897738; doi:10.3389/fphar.2018.00323)
Supplement: Supplementary file 1 [file Table_1.DOCX]

**Evidence supports tradition:
the *in vitro* effects of Roman chamomile on smooth muscles**

**Zsolt Sándor^1,±^, Javad Mottaghipisheh^2,±^, Katalin Veres^2^, Judit Hohmann^2^, Tímea Bencsik^3^, Attila Horváth^2^, Dezső Kelemen^4^, Róbert Papp^4^, Loránd Barthó^1^, Dezső Csupor^2,*^**

^1^Department of Pharmacology and Pharmacotherapy, University of Pécs, Medical School, Pécs, Hungary

^2^Department of Pharmacognosy, University of Szeged, Szeged, Hungary

^3^Department of Pharmacognosy, University of Pécs, Pécs, Hungary

^4^Department of Surgery, University of Pécs, Medical School, Clinical Center, Pécs, Hungary

**Correspondence:**

Dezső Csupor
[csupor.dezso@pharm.u-szeged.hu](mailto:csupor.dezso@pharm.u-szeged.hu)

**^±^** ZS and JM contributed equally to this work and both should be regarded as first authors.

**Content**

**Figure S1.** ^1^H-NMR Spectrum of Eupafolin (AN-2) (500 MHz, CD_3_OD) Page 3

**Figure S2.** JMOD Spectrum of Eupafolin (AN-2) (125 MHz, CD_3_OD) Page 3

**Figure S3.** ^1^H-NMR Spectrum of Hispidulin (CN-3) (500 MHz, CD_3_OD) Page 4

**Figure S4.** ^1^H-NMR Spectrum of Apigenin (CN-7) (500 MHz, CD_3_OD) Page 4

**Figure S5.** JMOD Spectrum of Apigenin (CN-7) (125 MHz, CD_3_OD) Page 5

**Figure S6.** ^1^H-NMR Spectrum of Luteolin (CN-8) (500 MHz, CD_3_OD) Page 5


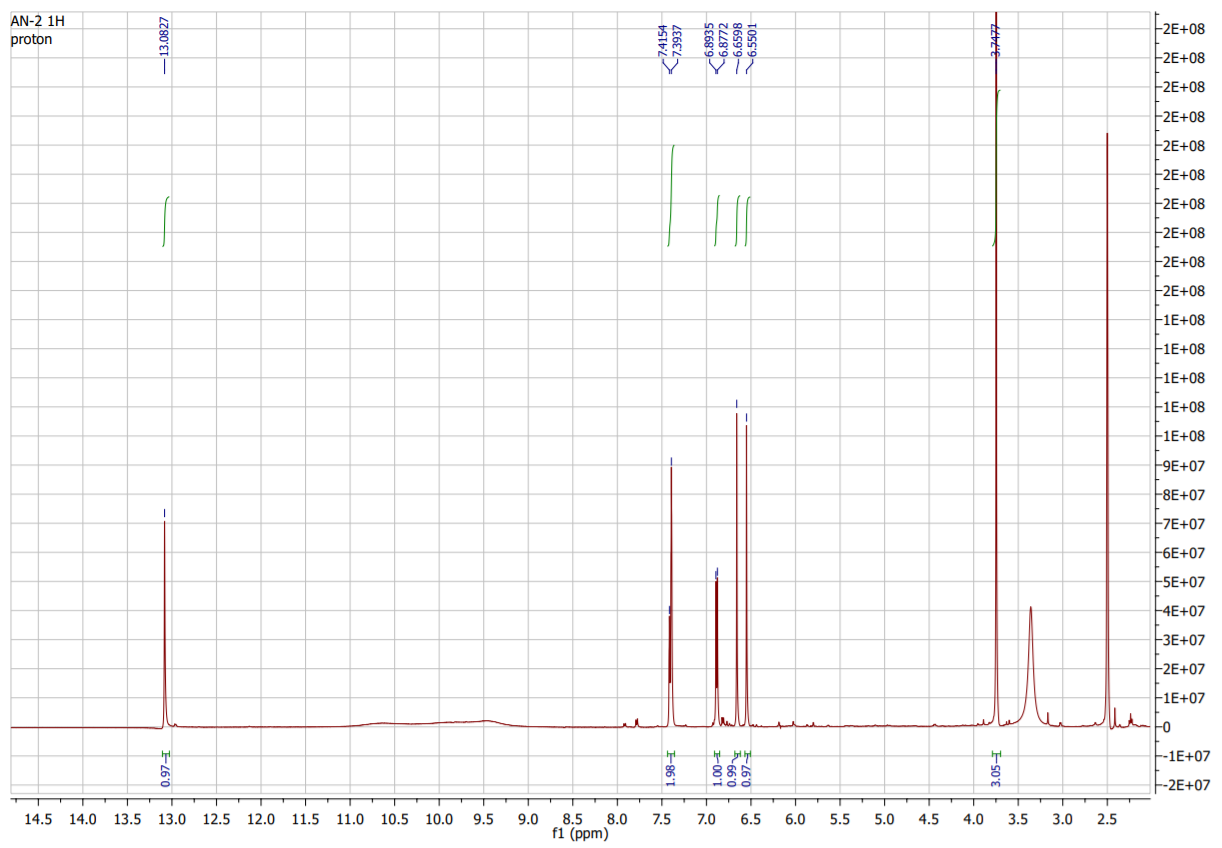


**Figure S1.** ^1^H-NMR Spectrum of Eupafolin (AN-2) (500 MHz, CD_3_OD)


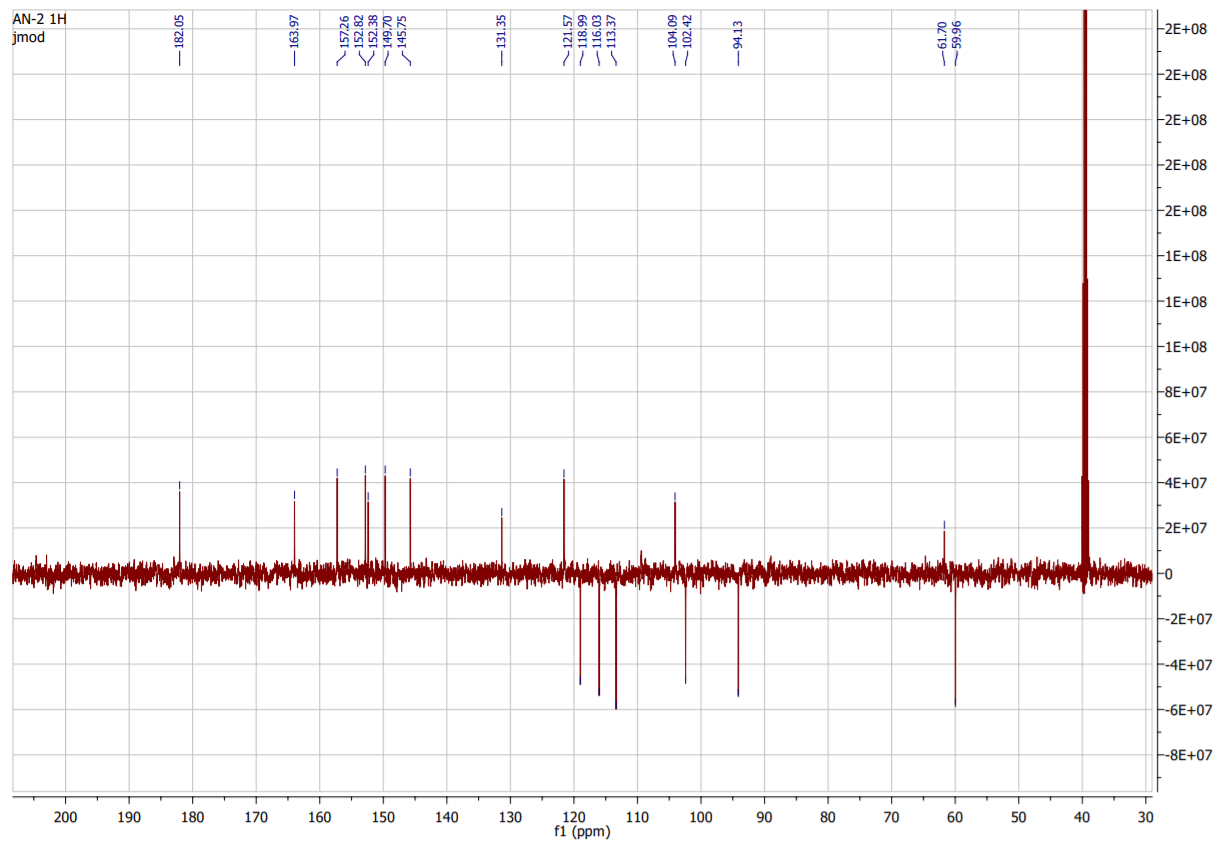


**Figure S2.** JMOD Spectrum of Eupafolin (AN-2) (125 MHz, CD_3_OD)


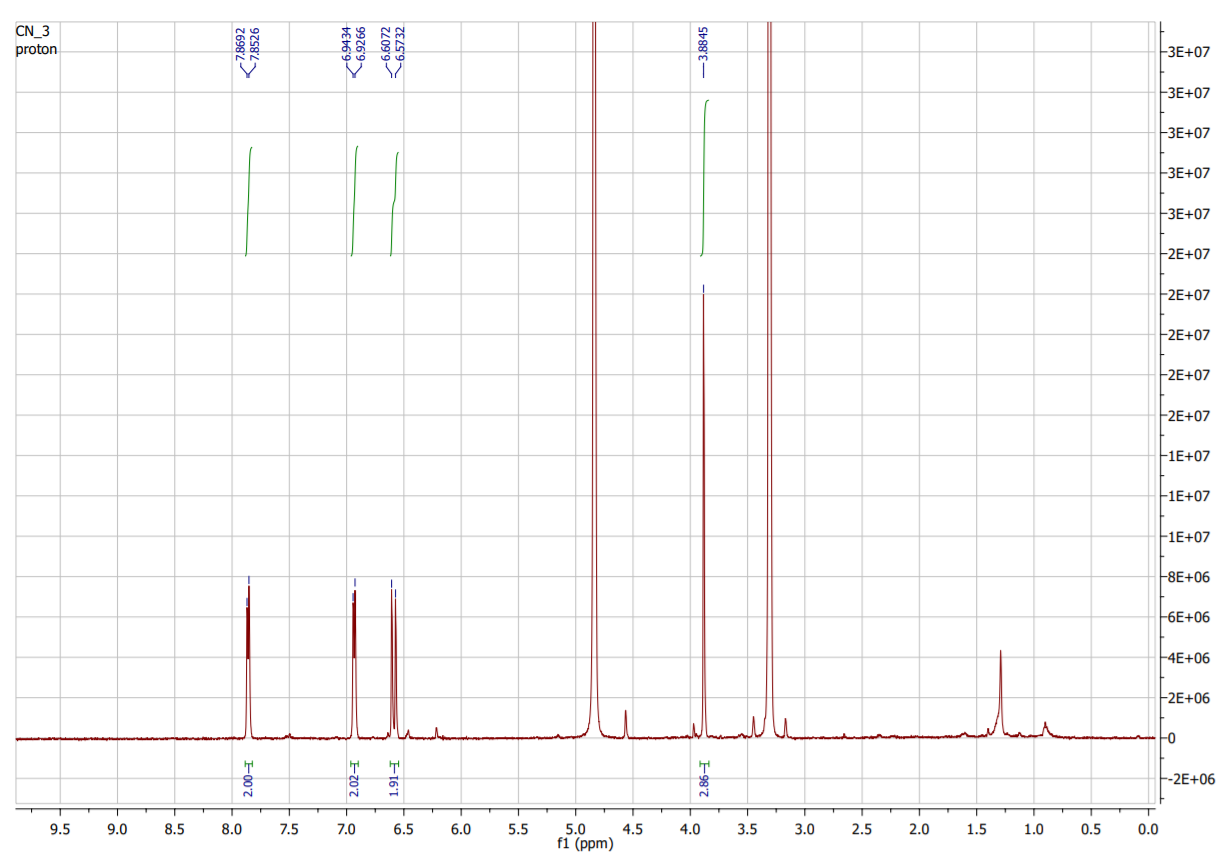


**Figure S3.** ^1^H-NMR Spectrum of Hispidulin (CN-3) (500 MHz, CD_3_OD)


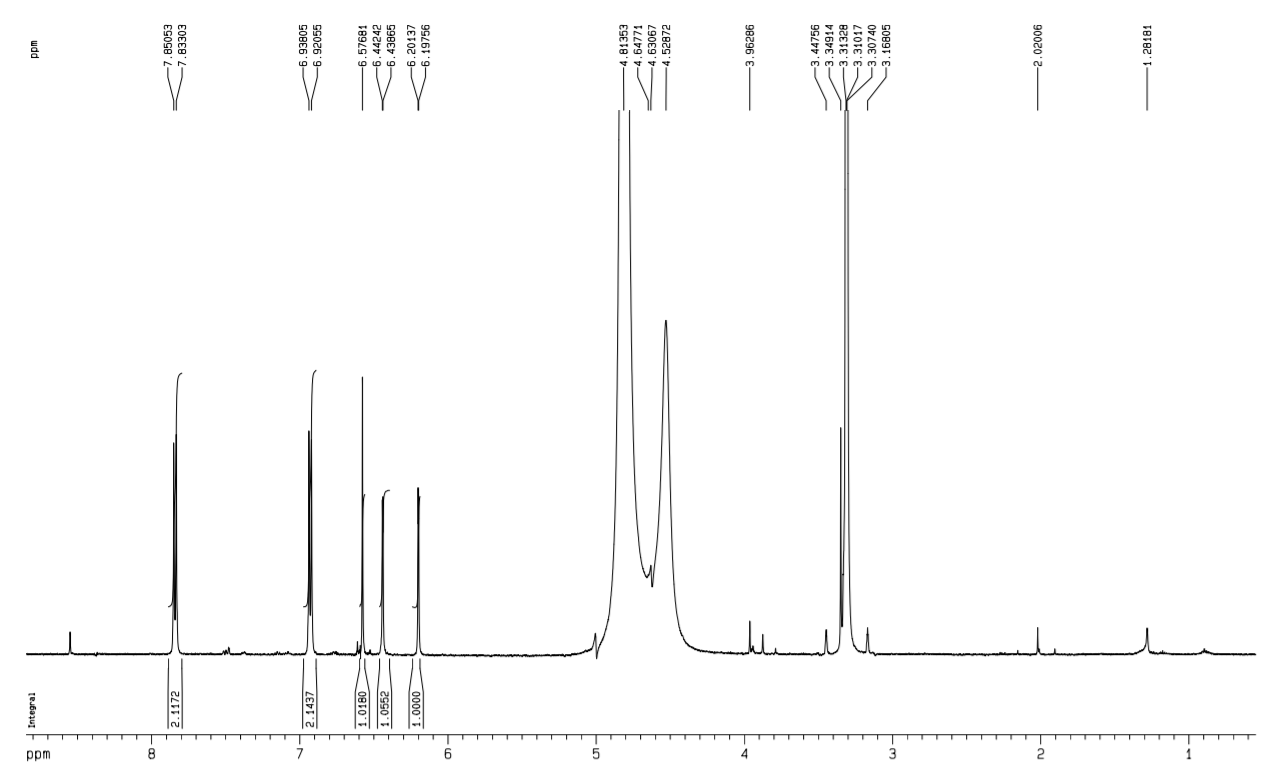


**Figure S4.** ^1^H-NMR Spectrum of Apigenin (CN-7) (500 MHz, CD_3_OD)


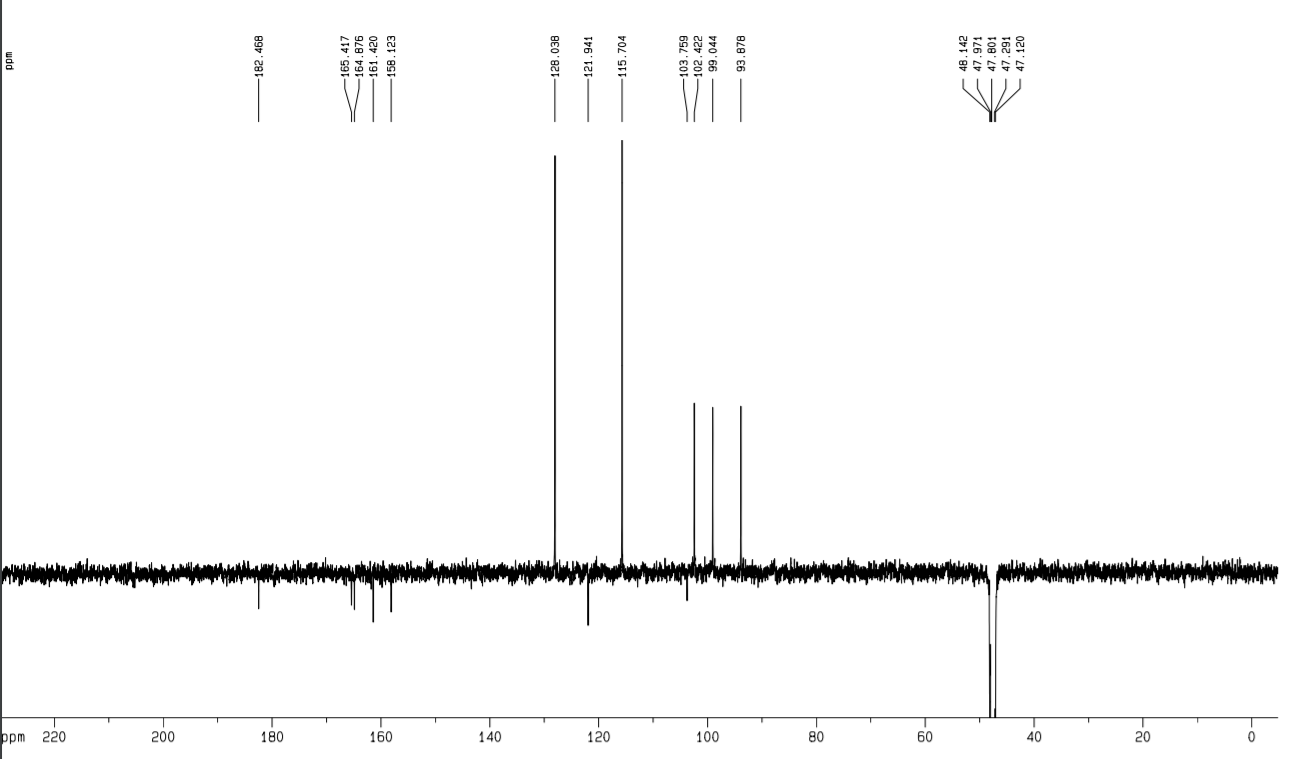


**Figure S5.** JMOD Spectrum of Apigenin (CN-7) (125 MHz, CD_3_OD)


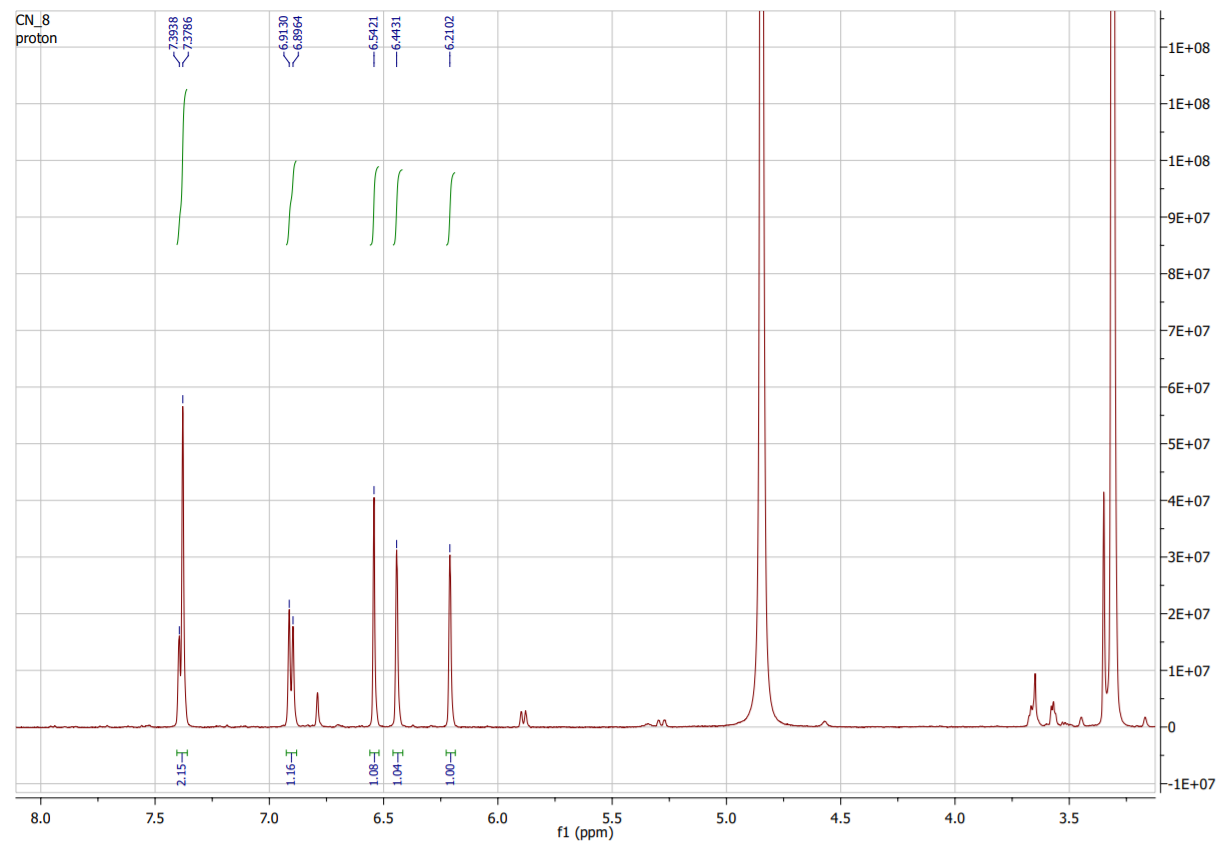


**Figure S6.** ^1^H-NMR Spectrum of Luteolin (CN-8) (500 MHz, CD_3_OD)
